# Predictive value of novel nutritional inflammation indexes in IVIG-unresponsive Kawasaki disease: a retrospective study

**DOI:** 10.3389/fnut.2025.1651750

**Published:** 2025-10-07

**Authors:** Cong Yi, Dan Xue, Jia Chen, Jun Guo, Yu-Neng Zhou, Yu Hu, Xiang She

**Affiliations:** Department of Pediatrics, Mianyang Central Hospital, School of Medicine, University of Electronic Science and Technology of China, Mianyang, China

**Keywords:** Kawasaki disease, C-reactive protein-albumin-lymphocyte index, C-reactive protein to albumin ratio, neutrophil-to-albumin ratio, neutrophil percentage-to-albumin ratio, prognostic nutritional index, intravenous immunoglobulin resistance

## Abstract

**Objective:**

This retrospective study aimed to investigate the predictive value of novel albumin-associated nutritional inflammation markers, including the C-reactive protein-albumin-lymphocyte (CALLY) index, C-reactive protein to albumin ratio (CAR), neutrophil-to-albumin ratio (NAR), neutrophil percentage-to-albumin ratio (NPAR) and prognostic nutritional index (PNI), for intravenous immunoglobulin (IVIG) resistance in Kawasaki disease (KD) patients.

**Methods:**

We conducted a retrospective analysis of clinical data from pediatric patients diagnosed with KD and admitted to our hospital between January 2012 and November 2023. Data were analyzed using univariate analysis, binary logistic regression analysis, and receiver operating curve (ROC) analysis.

**Results:**

The study included 716 children with KD, and 78 of them (10.9%) were diagnosed with IVIG-resistant KD. CAR, NAR and NPAR were positively correlated with IVIG resistance, while the CALLY index and PNI showed negative correlations. The area under the ROC curve (AUC) values of the CALLY index, CAR, NAR, NPAR and PNI were 0.725, 0.700, 0.580, 0.717 and 0.712.

**Conclusion:**

Our findings suggest that these novel nutritional inflammation indexes could be useful tools for predicting IVIG resistance in KD patients, potentially guiding timely intensified therapy. Compared to NAR, the CALLY index, CAR, NPAR, and PNI demonstrate stronger predictive performance for intravenous immunoglobulin (IVIG) resistance in Kawasaki disease and may hold greater potential for clinical application.

## 1 Introduction

Kawasaki disease (KD) is a systemic vasculitis primarily affecting medium-sized arteries, especially the coronary arteries, and is a major cause of acquired heart disease in children in developed countries (1, 2). Combining high-dose intravenous immunoglobulin (IVIG) therapy with aspirin has been established as the first-line therapy for KD and can effectively reduce the occurrence rate of coronary artery lesions (CALs), from 20 to 25% to approximately 2–4% (3). Nevertheless, about 10–20% of KD patients are unresponsive to IVIG therapy, increasing their risk of CALs (4). Thus, early prediction of IVIG resistance is crucial, as these patients may benefit from prompt intensified therapy.

While the cause of KD is not yet understood, systemic inflammatory responses are pivotal in its pathogenesis and progression. Albumin, once primarily seen as an indicator of nutritional status, is now also recognized as a protein in the acute inflammatory response (5). Increasing evidence highlights the interconnection between inflammation and nutrition. Serum albumin levels were found to have a negative correlation with inflammation. Studies have indicated that hypoalbuminemia is commonly observed in patients with KD during the acute phase, primarily resulting from increased vascular permeability and serum albumin leakage, highlighting changes in albumin-derived markers (6–9). In recent years, several novel inflammatory and nutritional indices have been proposed, including the C-reactive protein-albumin-lymphocyte (CALLY) index, C-reactive protein to albumin ratio (CAR), neutrophil-to-albumin ratio (NAR), neutrophil percentage-to-albumin ratio (NPAR) and prognostic nutritional index (PNI). These markers integrate multiple clinical evaluation parameters and provide more valuable information than individual markers alone (10–12). These inflammatory factors were initially mostly used to assess the prognosis of various cancers. Such as in 2021, the CALLY index was first applied to the prognosis research of hepatocellular carcinoma, and its correlation was confirmed (13). In 2016, NPAR was first applied to assess the prognosis of rectal cancer (14). Currently, various albumin-derived nutritional inflammation indices have been linked to the risk and severity of several diseases, such as tumors, as well as neurological, gastrointestinal, and respiratory diseases (15–18). Their affordability and widespread accessibility in daily clinical practice have increased their popularity, as a higher ratio frequently suggests a poor prognosis. However, to our knowledge, there are few studies on CAR, NPAR, and PNI, and no studies on the CALLY index and NAR in KD patients. While prior studies have examined individual inflammatory markers (e.g., CRP), this is the first to evaluate composite nutritional-inflammatory indexes (CALLY, CAR, NAR, NPAR, and PNI) in predicting IVIG resistance. Therefore, our study aimed to determine if albumin-derived markers (CALLY index, CAR, NAR, NPAR, and PNI) integrating inflammation and nutrition could be the significant predictors for IVIG resistance in KD patients.

## 2 Patients and methods

### 2.1 Participants

We retrospectively reviewed the clinical records of 716 pediatric patients with KD hospitalized at Mianyang Central Hospital between January 2012 and November 2023. Two experienced pediatricians, including at least one KD specialist, confirmed the diagnosis of complete and incomplete KD as per the 2017 American Heart Association guidelines (1). Two experienced pediatricians which with more than 5 years of clinical experience in pediatrics independently assessed each case. If both pediatricians reached the same conclusion, the patient was either included or excluded accordingly. In cases of disagreement, a third pediatrician was consulted to make the final determination. Complete KD diagnosis required ≥5 days of fever and ≥4 of the following clinical features: oral changes, extremity changes, rash, cervical lymphadenopathy, and bilateral bulbar conjunctival injection without exudate. Incomplete KD diagnosis included prolonged unexplained fever, <4 principal clinical features, and supportive laboratory or echocardiographic findings. All patients received IVIG (2 g/kg) intravenously and aspirin (30–50 mg/kg) orally. IVIG resistance was characterized by a persistent fever exceeding 38 °C at 36 h post-initial IVIG dose, or a recurrent fever accompanied by at least one primary clinical symptom of KD within 2 weeks, typically between 2 and 7 days post-treatment. KD shock syndrome (KDSS) was characterized by a ≥ 20% decrease in baseline systolic blood pressure or clinical signs of hypoperfusion (19).

Inclusion criteria included: (1) confirmed diagnosis of KD; (2) patients with age of 28 days or more and 16 years or less. Exclusion criteria included: (1) patients without IVIG therapy during hospitalization; (2) patients lacking complete data; (3) patients treated with glucocorticoid, other immunosuppressive drugs, or IVIG at other medical facilities; (4) patients with recurrent KD.

Data regarding the demographics, clinical indicators, and laboratory parameters were obtained from the hospital’s electronic records. All laboratory indicators were collected for assessment during the acute febrile phase prior to IVIG treatment. In line with previous studies (10, 16–18, 20), we calculated the CALLY index, CAR, NAR, NPAR and PNI using the following formulas:

CALLY index = albumin × lymphocyte count/(CRP × 10).

CAR = CRP/albumin.

NAR = neutrophil count/albumin.

NPAR = neutrophil percentage/albumin.

PNI = albumin+5 × lymphocyte count.

The Ethics Committee of Mianyang Central Hospital approved this study, with informed consent being waived (No. S20250312-01). This study was in line with the Helsinki declaration.

### 2.2 Statistical analysis

Normally distributed continuous variables are presented as mean ± standard deviation (SD) and analyzed between groups using Student’s *t*-test. Non-normally distributed variables are expressed as median (interquartile range, IQR) and compared between groups using the Mann–Whitney *U* test. Categorical data are presented as numbers (%) and analyzed using the Chi-square test or Fisher’s exact test. Binary logistic regression analysis was employed for conducting multivariate analysis by using variables of *p* < 0.1 in univariate analysis. To avoid collinearity, albumin-based markers were incorporated and assessed in the separate statistical models. The predictive ability of each novel nutritional inflammation marker for identifying IVIG resistance was assessed by the receiver operating characteristic (ROC) curve. Statistical analyses were conducted with SPSS 22.0, considering a *p*-value <0.05 as significant.

## 3 Results

**Figure 1:**
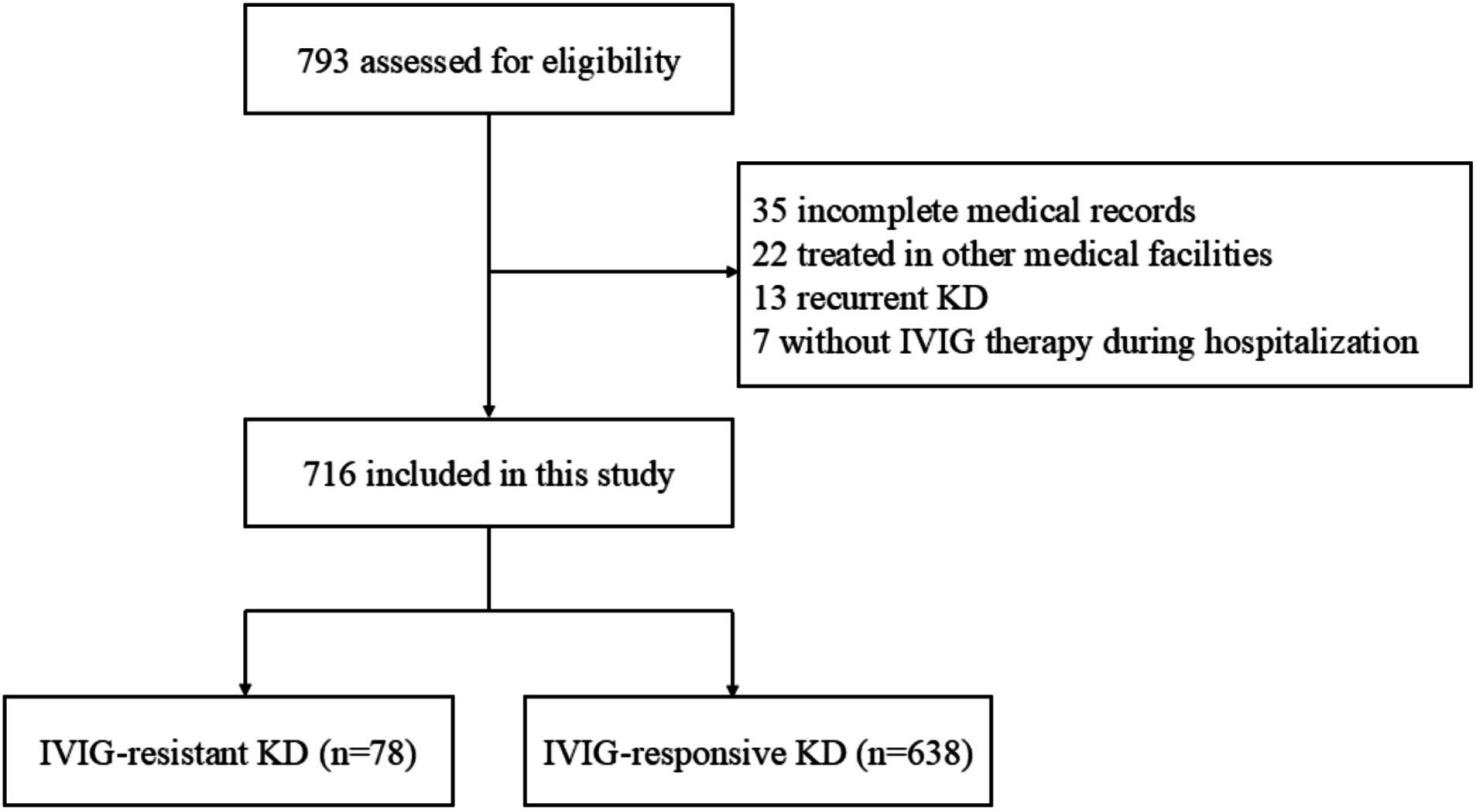
Flowchart of patient selection.

After excluding 77 cases based on the predefined exclusion criteria (Figure 1), 716 patients with KD were enrolled, consisting of 429 (59.9%) males and 287 (40.1%) females. The median age was 2.2 (1.2, 3.7) years old. Among the included individuals, 78 (10.9%) and 638 (89.1%) were diagnosed with IVIG-resistant KD and IVIG-responsive KD, respectively.

As shown in Table 1, no significant differences were observed between the IVIG-responsive group and IVIG-resistant group regarding sex, age, the occurrence of incomplete KD, and the other four typical clinical manifestations of KD, except extremity changes (*p* > 0.05). The IVIG-resistant group showed a significantly higher incidence of extremity changes, irritability, jaundice, tachypnea, aseptic meningitis, and KDSS than the IVIG-responsive group (all *p* < 0.05).

**Table 1:** Clinical characteristics of KD patients with IVIG resistance and IVIG response.

Variables	IVIG-resistance	IVIG-response	*p*-value
Patients, *n*	78	638	–
Age (year), median (IQR)	2.5 (1.2–3.9)	2.1 (1.2–3.7)	0.495
Gender, male, *n* (%)	43(55.1)	386(60.5)	0.361
Days of IVIG at initiation, mean ± SD	6.0 ± 1.7	6.6 ± 1.6	0.001
Days of IVIG at initiation < 5 days, *n* (%)	8 (10.3)	18 (2.8)	0.006
Days of IVIG at initiation > 10 days, *n* (%)	2 (2.6)	25 (3.9)	0.781
Fever, *n* (%)	78(100)	638(100)	1.000
Conjunctival injection, *n* (%)	69(88.5)	581(90.1)	0.453
Rash, *n* (%)	65(83.3)	505(79.2)	0.660
Oral mucosal changes, *n* (%)	68(87.2)	560(87.8)	0.880
Extremity changes, *n* (%)	63(80.8)	430(67.4)	0.016
Cervical lymphadenopathy, *n* (%)	54(69.2)	456(71.5)	0.680
Vomiting, *n* (%)	17(21.8)	100(15.7)	0.366
Diarrhea, *n* (%)	19(24.4)	155(24.3)	0.990
Jaundice, *n* (%)	7(9.0)	21(3.3)	0.015
Cough, *n* (%)	50(64.1)	380(59.6)	0.440
Expectoration, *n* (%)	30(38.5)	178(27.9)	0.146
Tachypnea, *n* (%)	15(19.2)	9(1.4)	<0.001
Irritability, *n* (%)	24(30.8)	72(11.3)	<0.001
Seizure, *n* (%)	0(0)	7(1.1)	1.000
Aseptic encephalitis, *n* (%)	9(11.5)	18(2.8)	<0.001
KDSS, *n* (%)	3(3.8)	2(0.3)	0.005
IKD, *n* (%)	11(14.1)	114(17.9)	0.408
Coronary artery lesion (CAL), *n* (%)	6 (7.7)	41 (6.4)	0.670
Kobayashi score >4, *n* (%)	17 (21.8)	37 (5.8)	<0.001

IKD, incomplete Kawasaki disease; IQR, interquartile range; IVIG, intravenous immunoglobulin; KD, Kawasaki disease; KDSS, KD shock syndrome; SD, standard deviation.

**Figure 2:**
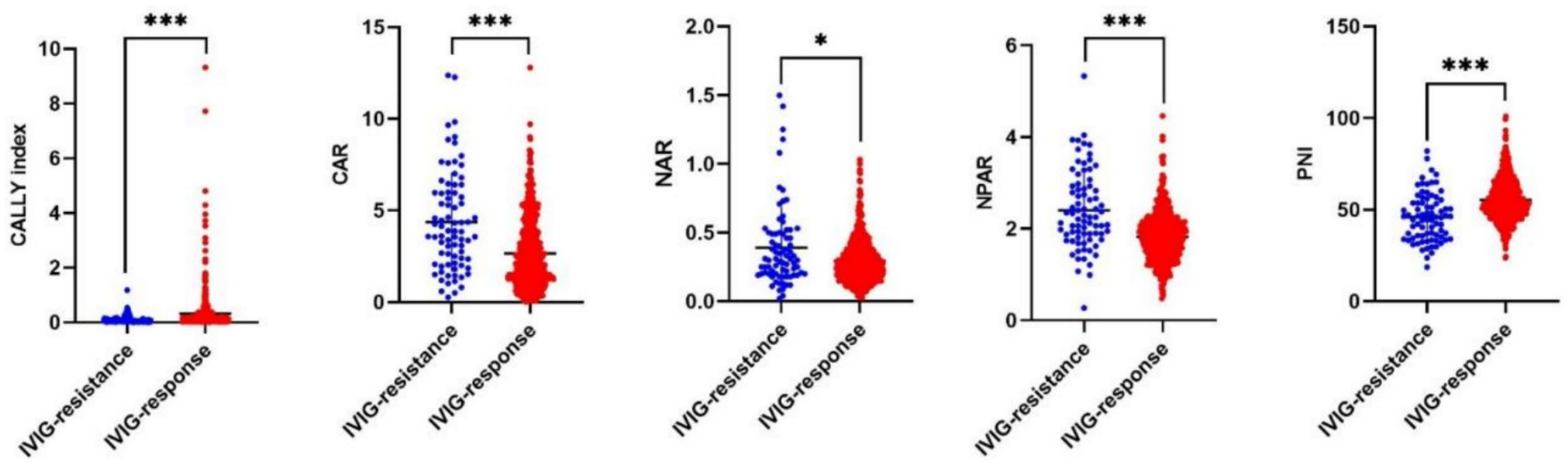
The distribution of 5 novel albumin-associated nutritional inflammation markers in KD patients with IVIG resistance and IVIG response. In IVIG-resistant group, CAR, NAR and NPAR were significantly higher, whereas the CALLY index and PNI were significantly lower compared to IVIG-responsive group.

The laboratory findings are shown in Table 2. The IVIG-resistant group exhibited notably elevated CRP, AST and bile acids levels, alongside reduced lymphocytes, hemoglobin, albumin and serum sodium levels compared to the IVIG-responsive group (all *p* < 0.05). These findings aligned with earlier studies (21–23). Furthermore, it was noted that in the IVIG-resistant group, CAR, NAR and NPAR were significantly higher, whereas the CALLY index and PNI were significantly lower compared to IVIG-responsive group (Table 2 and Figure 2). However, the other laboratory parameters did not differ significantly between the two groups (all *p* > 0.05).

**Table 2:** Laboratory findings of KD patients with IVIG resistance and IVIG response.

Variables	IVIG-resistance	IVIG-response	*p*-value
WBC (×10^9^/L), mean ± SD	15.65 ± 8.25	15.76 ± 5.23	0.905
Neutrophils (×10^9^/L), mean ± SD	11.82 ± 7.33	10.86 ± 5.23	0.266
Neutrophil percentage, mean ± SD	0.73 ± 0.15	0.67 ± 0.14	0.001
Lymphocytes (×10^9^/L), mean ± SD	2.66 ± 1.89	3.50 ± 1.90	0.001
Monocytes (×10^9^/L), mean ± SD	1.02 ± 0.97	1.06 ± 0.59	0.737
Platelet (×10^9^/L), mean ± SD	331.9 ± 211.9	343.3 ± 138.1	0.646
Hemoglobin (g/L), mean ± SD	101.4 ± 14.3	111.4 ± 12.8	<0.001
CRP (mg/L), median (IQR)	132.4(78.9–158.4)	83.2(50.0–134.2)	<0.001
ESR (mm/h), median (IQR)	53.0(34.8–77.6)	57.5(40.0–76.0)	0.616
ALT (IU/L), median (IQR)	33(17–83)	25(15–82)	0.236
AST (IU/L), median (IQR)	40(27–67)	32(25–48)	0.042
Bile acids (mmol/L), median (IQR)	8.2(4.9–15.3)	5.9(4.2–8.7)	<0.001
Serum sodium (mmol/L), mean ± SD	134.8 ± 3.6	136.8 ± 3.0	<0.001
Albumin (g/L), mean ± SD	32.67 ± 7.49	37.87 ± 4.91	<0.001
CALLY index, median (IQR)	0.06 (0.03–0.13)	0.15 (0.07–0.31)	<0.001
CAR, median (IQR)	3.86(2.12–5.98)	2.22(1.26–3.73)	<0.001
PNI, mean ± SD	45.95 ± 13.04	55.30 ± 11.68	<0.001
NAR, median (IQR)	0.31(0.20–0.49)	0.27(0.19–0.37)	0.021
NPAR, mean ± SD	2.40 ± 0.86	1.82 ± 0.52	<0.001

ALT, alanine transaminase; AST, aspartate transaminase; CALLY, C-reactive protein-Albumin-lymphocyte; CAR, C-reactive protein to albumin ratio; CRP, C-reactive proteins; ESR, erythrocyte sediment rate; IQR, interquartile range; IVIG, intravenous immunoglobulin; NAR, neutrophil-to-albumin ratio; NPAR, neutrophil percentage-to-albumin ratio; PNI, prognostic nutritional index; SD, standard deviation; WBC, white blood cell count.

This study investigated the potential role of albumin-based biomarkers in contributing to IVIG responsiveness in KD. The potential predictive values of albumin-based markers including the CALLY index, CAR, NAR, NPAR and PNI, which were shown to be different between the two groups, were assessed in the models. To avoid collinearity, albumin-based markers were incorporated and assessed in the separate statistical models (Supplementary Tables 1–5), Among all the models, variables of Days of IVIG at initiation, Tachypnea, Hemoglobin, and Serum sodium were statistically significant in the binary logistic regression analysis. Therefore, we included the four variables to construct the Base Model, while Models 1–5 were, respectively, formed by incorporating CALLY index, CAR, NAR, NPAR and PNI into the Base Model to compare whether the predictive value of the model increased after the inclusion of these novel nutritional inflammation indexes, respectively. The CALLY index (OR: 0.072, 95% CI: 0.009–0.577, *p* = 0.013), CAR (OR: 1.207, 95% CI: 1.070–1.361, *p* = 0.002), NAR (OR: 6.073, 95% CI: 1.610–22.916, *p* = 0.008), NPAR (OR: 2.535, 95% CI: 1.648–3.898, *p* < 0.001), and PNI (OR: 0.953, 95% CI: 0.927–0.979, *p* = 0.001) were independent predictors for IVIG-resistance (Table 3, the brief table and Supplementary Table 6, the complete table).

**Table 3:** Binary logistic regression analysis to evaluate risk factors for IVIG resistance in different models.

Models	Variables	*B*	S. E.	Wald *χ*^2^	OR	95%CI	*p*-value
Base model	Days of IVIG at initiation	−0.287	0.095	9.043	0.751	0.622–0.905	0.003
	Tachypnea	1.933	0.517	13.986	6.908	2.509–19.023	<0.001
	Hemoglobin	−0.055	0.010	28.929	0.946	0.927–0.966	<0.001
	Serum sodium	−0.156	0.041	14.562	0.855	0.789–0.927	<0.001
Model 1	CALLY index	−2.632	1.062	6.143	0.072	0.009–0.577	0.013
Model 2	CAR	0.188	0.061	9.377	1.207	1.070–1.361	0.002
Model 3	NAR	1.804	0.678	7.089	6.073	1.610–22.916	0.008
Model 4	NPAR	0.930	0.220	17.949	2.535	1.648–3.898	<0.001
Model 5	PNI	−0.051	0.013	15.717	0.950	0.927–0.975	<0.001

CALLY, C-reactive protein-albumin-lymphocyte; CAR, C-reactive protein to albumin ratio; CI, confidence interval; IVIG, intravenous immunoglobulin; NAR, neutrophil-to-albumin ratio; NPAR, neutrophil percentage-to-albumin ratio; PNI, prognostic nutritional index.

**Figure 3:**
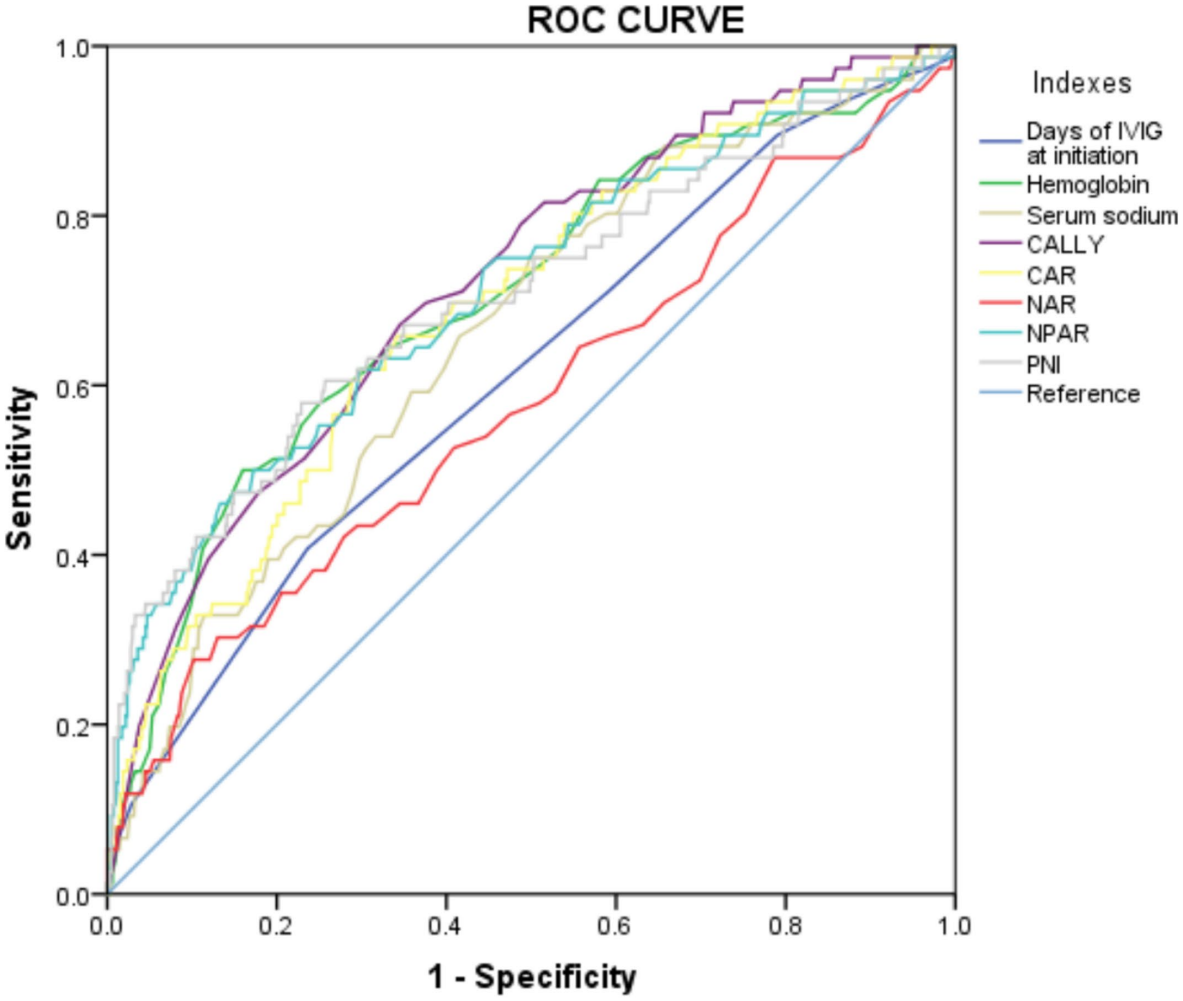
The ROC curve for models in predicting IVIG resistance in the whole cohort. CALLY, C-reactive protein-albumin-lymphocyte; CAR, C-reactive protein to albumin ratio; NAR, neutrophil-to-albumin ratio; NPAR, neutrophil percentage-to-albumin ratio; PNI, prognostic nutritional index.

**Figure 4:**
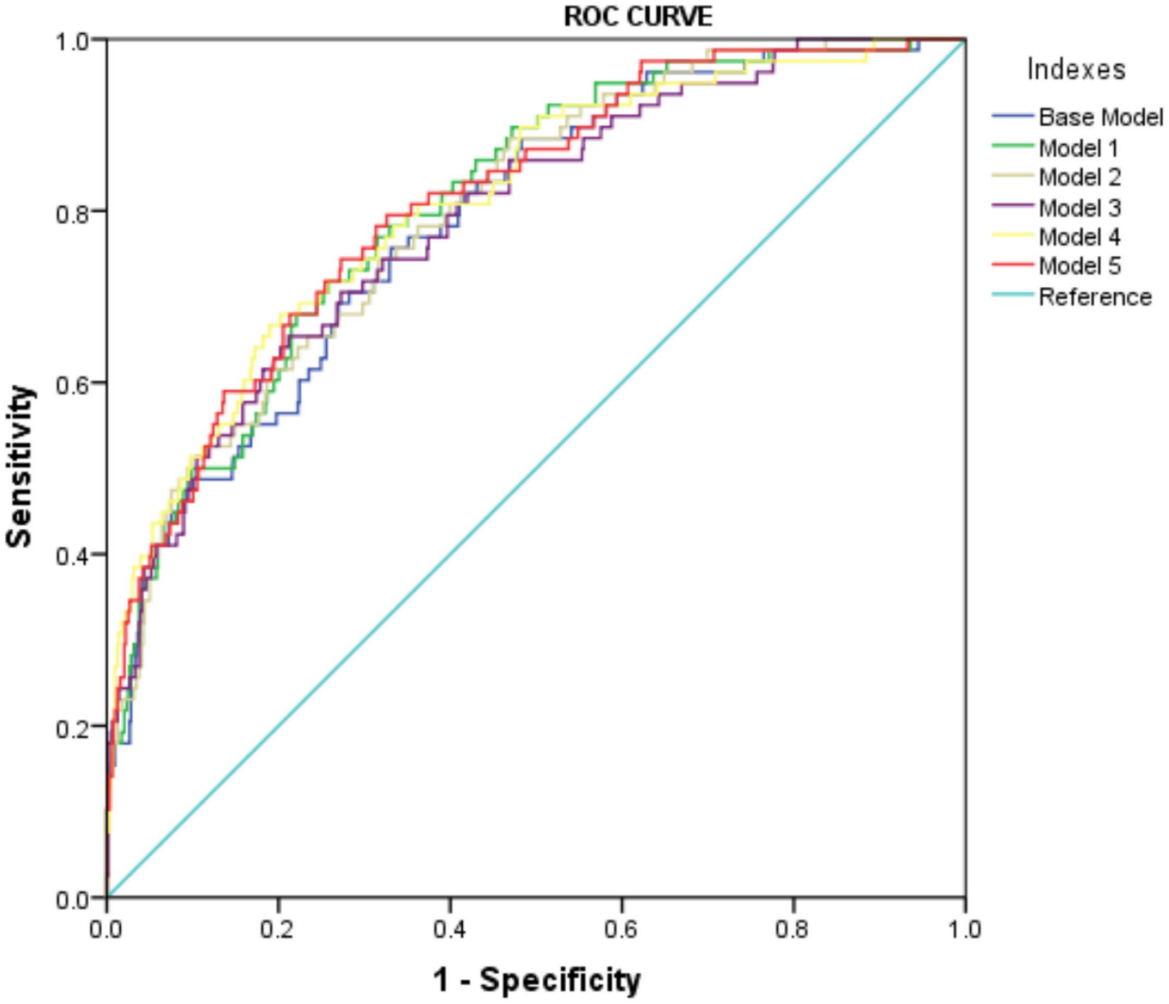
The ROC curve of predicted probabilities in different Models in predicting IVIG resistance.

Furthermore, the predictive power of those above albumin-derived markers for IVIG-resistance were analyzed (Tables 4, 5; Figure 3; Supplementary Tables 7–11). ROC curve analyses indicated that the CALLY index, CAR, NAR, NPAR, and PNI are predictive of IVIG-resistance, with AUC values of 0.725 (cut-off value: 0.105, sensitivity: 66.4%, specificity: 70.5%), 0.700 (cut-off value: 3.245, sensitivity: 62.8%, specificity: 69.6%), 0.580 (cut-off value: 0.485, sensitivity: 28.2%, specificity: 89.8%), 0.717 (cut-off value: 2.335, sensitivity: 47.4%, specificity: 88.7%), and 0.712 (cut-off value: 47.255, sensitivity: 42.3%, specificity: 22.7%), respectively. Meanwhile, the AUC values corresponding to the multiple comparison analysis of the predicted probabilities of each model was presented (Table 6 and Figure 4). As shown in Table 6, the AUC values of Models 1–5 have increased compared to those of the Base Model. However, the DeLong test results indicate that there was no statistically significant difference in AUC between the Base Model and Models 1–5 (Table 7).

**Table 4:** Area under the curve of CALLY index, CAR, NAR, NPAR, and PNI in different models.

Models	Variables	AUC	95%CI	*p*-value
Model 1	CALLY index	0.725	0.664–0.785	<0.001
Model 2	CAR	0.700	0.637–0.763	<0.001
Model 3	NAR	0.580	0.506–0.654	0.038
Model 4	NPAR	0.717	0.651–0.784	<0.001
Model 5	PNI	0.712	0.643–0.788	<0.001

AUC, area under curve; CI, confidence interval; CALLY, C-reactive protein-albumin-lymphocyte; CAR, C-reactive protein to albumin ratio; NAR, neutrophil-to-albumin ratio; NPAR, neutrophil percentage-to-albumin ratio; PNI, prognostic nutritional index.

**Table 5:** The ROC curve for variables’ levels in predicting IVIG resistance in the whole cohort.

Models	Variables	Cutoff	Sensitivity (%)	Specificity (%)
Model 1	CALLY	9917.65	66.4	70.5
Model 2	CAR	3.245	62.8	69.6
Model 3	NAR	0.485	28.2	89.8
Model 4	NPAR	2.335	47.4	88.7
Model 5	PNI	47.255	42.3	22.7
All models	Days of IVIG at initiation	5.5	59.0	23.7
All models	Hemoglobin	98.5	50.0	16.0
All models	Serum sodium	136.95	24.4	49.8

CALLY, C-reactive protein-albumin-lymphocyte; CAR, C-reactive protein to albumin ratio; IVIG, intravenous immunoglobulin; NAR, neutrophil-to-albumin ratio; NPAR, neutrophil percentage-to-albumin ratio; PNI, prognostic nutritional index; ROC, receiver operating curve.

**Table 6:** Area under the curve of predicted probabilities in different models.

Models	AUC	95%CI	*p*-value
Base model	0.789	0.736–0.841	<0.001
Model 1	0.806	0.757–0.856	<0.001
Model 2	0.800	0.750–0.850	<0.001
Model 3	0.791	0.737–0.845	<0.001
Model 4	0.810	0.757–0.862	<0.001
Model 5	0.811	0.760–0.861	<0.001

**Table 7:** The AUC differences between the Base Model and Models 1–5 under the DeLong test.

Comparison	The difference in AUC	*p*-value	95%CI lower limit	95%CI upper limit
Base Model-Model 1	−0.018	0.157	−0.043	0.007
Base Model-Model 2	−0.011	0.316	−0.033	0.011
Base Model-Model 3	−0.002	0.799	−0.021	0.017
Base Model-Model 4	−0.021	0.176	−0.052	0.009
Base Model-Model 5	−0.022	0.167	−0.054	0.009

## 4 Discussion

Research on IVIG-resistant KD has long been a significant challenge for pediatricians. Over the past decades, efforts have been made to identify more effective methods for predicting IVIG resistance. Initially, single factors such as CRP and albumin were used; later, scoring systems incorporating multiple variables, such as the Kobayashi score, were developed. While single biomarkers lack comprehensiveness in capturing the complex inflammatory response during the acute phase of KD, multi-factor scoring systems can be somewhat cumbersome in clinical settings. Therefore, this study aims to explore inflammatory indicators that are both comprehensive and clinically practical for predicting IVIG-resistant KD. This was a relatively large-scale study and, to our knowledge, the first to explore albumin-related biomarkers, including the CALLY index, CAR, NAR, NPAR and PNI, in IVIG-resistant KD. In our study we compared albumin-derived nutritional inflammation indices between two groups and found that CAR, NAR and NPAR were positively correlated with IVIG resistance, while the CALLY index and PNI showed negative correlations. In conclusion, our findings suggest that the CALLY index, CAR, NAR, NPAR and PNI are associated with IVIG resistance risk.

Serum albumin produced by the liver is a negative acute-phase reactant (AFR) that decreases during immune activation, whereas CRP produced by the liver is a positive AFR that increases in inflammation or infection. Neutrophils indicate active inflammation, whereas lymphocytes serve as markers for immune regulation. During an inflammatory response, delayed neutrophil apoptosis and stem cell stimulation by growth factors result in neutrophilia and redistribution within the lymphatic system, while increased lymphocyte apoptosis causes lymphocytopenia (24). Consistent with these findings, our study demonstrated that the IVIG-resistant group exhibited elevated CRP levels and reduced lymphocyte and albumin levels compared to the IVIG-responsive group. The absence of consensus on a singular risk factor and the susceptibility of individual inflammatory parameters to external influences have led to a research focus on combined risk factor indices. These indices are considered potentially more reliable for predicting IVIG resistance in KD patients than individual parameters.

Albumin-derived markers (CALLY index, CAR, NAR, NPAR, and PNI), calculated based on serum albumin, CRP, lymphocyte count, and neutrophils, more comprehensively reflect the nutritional and inflammation status. These markers are crucial for evaluating the severity and predicting outcomes of inflammatory conditions (25–27). The CALLY index, introduced by Liu et al. (28), combines albumin, lymphocyte count and CRP, offering insights into nutritional status, inflammation, and immune function (14). NAR serves as a crucial index that integrates the advantages of neutrophils and albumin to provide a comprehensive assessment of systemic immunity and nutritional status. This study represents the inaugural examination of the CALLY index and NAR within the framework of KD. In our study, we found that the results of the ROC analysis demonstrated that the CALLY index had the highest AUC among the parameters evaluated, indicating that it exhibited superior diagnostic performance. However, NAR demonstrated the least effective performance in predicting IVIG resistance in KD, as indicated by its lowest AUC among the evaluated parameters. It was well recognized that during the acute phase of KD serum albumin levels and lymphocyte counts were typically reduced, whereas CRP levels, neutrophil counts, and the neutrophil percentage were elevated. Among these albumin-derived markers, the three components of the CALLY Indexes show a consistent direction of change, while the other four albumin-derived markers show a consistent direction of change for two of their components. This might be the reason why the CALLY Indexes have the highest predictive value. In the univariate analysis of the components of the five albumin-derived markers, albumin levels, lymphocyte count, CRP levels, and neutrophil percentage demonstrated statistically significant differences between the two patient groups, whereas neutrophil count did not reach statistical significance. This might partially explain the relatively lower predictive value of NAR.

To date, limited research has explored the association between CAR, NPAR, PNI, and KD. A meta-analysis showed that lower PNI or high CAR was associated with the increased risk to develop IVIG resistance (28). NPAR was identified an independent biomarker for IVIG resistance (10). Our findings confirm that CAR, NPAR, and PNI are independent risk factors for IVIG resistance, aligning with previous research. Unlike previous studies, which often analyzed these markers individually, our study comprehensively examined the association of these albumin-derived markers with IVIG-resistant KD. As of the time of writing this manuscript, our study was the first to report the association between CALLY indexes and IVIG-resistant KD. Additionally, only one previous study had investigated the predictive value of NPAR for IVIG-resistant KD, and it was based on a sample size of 438 cases, which is considerably smaller than that of our study (10). This study indicates that PNI, NPAR, and CAR are potential predictive factors for IVIG resistance in KD, a finding that aligns with the results of previous research. In this study, we also calculated the Kobayashi score for each patient. However, its sensitivity for identifying IVIG resistance in Kawasaki disease was only 21.8% (17/78), which was lower than that of other albumin-derived markers evaluated in this study. Therefore, in this study, compared with the Kobayashi score, the albumin-derived markers demonstrate greater potential for clinical application.

This study has certain limitations. First, owing to its retrospective, single-center design and the insufficient sample size of patients, and 35 patients were excluded from the study due to missing data, all of which inevitably led to a certain degree of selection bias in our research process. The potential for bias cannot be completely ruled out, which may reduce the statistical power of our findings. Second, the homogeneity of the study population in terms of racial background may limit the generalizability of our findings. For instance, the Kobayashi score, which has achieved relatively good predictive effects in previous reports, may not perform well in our study due to differences in ethnicity. Therefore, prospective multicenter studies with larger and more diverse racial samples are necessary to confirm these results. The association of hypoalbuminemia (PNI, CAR) with IVIG resistance aligns with KD’s endothelial dysfunction paradigm. For future work, we need to proactively collect data on KD patients in our hospital or from other centers for external validation, so that our research can be better applied in clinical practice.

## Data Availability

The original contributions presented in the study are included in the article/Supplementary material, further inquiries can be directed to the corresponding authors.
