# Supplementary material for: Predictive value of novel nutritional inflammation indexes in IVIG-unresponsive Kawasaki disease: a retrospective study
Source: Front Nutr. 2025 Oct 7;12:1651750. doi: 10.3389/fnut.2025.1651750 (PMC12537436; doi:10.3389/fnut.2025.1651750)
Supplement: Supplementary file 1 [file Table_1.docx]

**Table 1** Binary logistic regression analysis to evaluate risk factors for IVIG resistance in Model 1.

| **Variables** | **B** | **S.E.** | **Waldχ2** | **OR** | **95%CI** | **p value** |
| --- | --- | --- | --- | --- | --- | --- |
| **Days of IVIG at initiation** | -0.253 | 0.098 | 6.583 | 0.777 | 0.640-0.942 | 0.010* |
| **Extremity changes** | 0.403 | 0.332 | 1.476 | 1.496 | 0.781-2.865 | 0.224 |
| **jaundice** | 0.225 | 0.738 | 0.093 | 1.252 | 0.295-5.320 | 0.761 |
| **Tachypnea** | 1.837 | 0.604 | 9.256 | 6.278 | 1.922-20.501 | 0.002* |
| **Irritability** | 0.203 | 0.388 | 0.274 | 1.225 | 0.573-2.621 | 0.601 |
| **Aseptic encephalitis** | 0.502 | 0.646 | 0.604 | 1.652 | 0.466-5.855 | 0.437 |
| **KD shock syndrome** | -0.929 | 1.221 | 0.580 | 0.395 | 0.036-4.320 | 0.446 |
| **Hemoglobin** | -0.052 | 0.011 | 23.077 | 0.950 | 0.930-0.970 | <0.001* |
| **AST** | <0.001 | 0.001 | 0.427 | 1.000 | 0.999-1.001 | 0.513 |
| **Bile acids** | 0.005 | 0.009 | 0.324 | 1.005 | 0.988-1.022 | 0.569 |
| **Serum sodium** | -0.122 | 0.045 | 7.277 | 0.885 | 0.810-0.967 | 0.007* |
| **CALLY** | -2.437 | 1.061 | 5.281 | 0.087 | 0.011-0.699 | 0.022* |

**Note:** *p < 0.05.

**Abbreviations:** AST, aspartate transaminase; KD, Kawasaki disease; CALLY, C-reactive protein-albumin-lymphocyte.

|  |
| --- |

**Table 2** Binary logistic regression analysis to evaluate risk factors for IVIG resistance in Model 2.

| **Variables** | **B** | **S.E.** | **Waldχ2** | **OR** | **95%CI** | **p value** |
| --- | --- | --- | --- | --- | --- | --- |
| **Days of IVIG at initiation** | -0.253 | 0.102 | 6.188 | 0.777 | 0.637-0.948 | 0.013* |
| **Extremity changes** | 0.407 | 0.333 | 1.491 | 1.502 | 0.782-2.888 | 0.222 |
| **jaundice** | 0.300 | 0.754 | 0.158 | 1.350 | 0.308-5.922 | 0.691 |
| **Tachypnea** | 1.711 | 0.612 | 7.810 | 5.533 | 1.667-18.366 | 0.005* |
| **Irritability** | 0.237 | 0.395 | 0.362 | 1.268 | 0.585-2.748 | 0.548 |
| **Aseptic encephalitis** | 0.510 | 0.638 | 0.637 | 1.665 | 0.476-5.818 | 0.425 |
| **KD shock syndrome** | -1.723 | 1.195 | 2.080 | 0.178 | 0.017-1.856 | 0.149 |
| **Lymphocytes** | -0.205 | 0.087 | 5.549 | 0.815 | 0.687-0.966 | 0.018* |
| **Hemoglobin** | -0.060 | 0.011 | 28.897 | 0.942 | 0.921-0.963 | <0.001* |
| **CRP** | 0.001 | 0.002 | 0.209 | 1.001 | 0.997-1.005 | 0.647 |
| **AST** | <0.001 | 0.001 | 0.796 | 1.000 | 0.999-1.001 | 0.372 |
| **Bile acids** | 0.003 | 0.009 | 0.122 | 1.003 | 0.986-1.021 | 0.727 |
| **Serum sodium** | -0.121 | 0.046 | 6.959 | 0.886 | 0.810-0.969 | 0.008* |
| **NAR** | 1.904 | 0.728 | 6.834 | 6.714 | 1.610-27.989 | 0.009* |

**Note:** *p < 0.05.

**Abbreviations:** AST, aspartate transaminase; CRP, C-reactive proteins; KD, Kawasaki disease; NAR, neutrophil-to-albumin ratio.

**Table 3** Binary logistic regression analysis to evaluate risk factors for IVIG resistance in Model 3.

| **Variables** | **B** | **S.E.** | **Waldχ2** | **OR** | **95%CI** | **p value** |
| --- | --- | --- | --- | --- | --- | --- |
| **Days of IVIG at initiation** | -0.264 | 0.100 | 6.950 | 0.768 | 0.631-0.935 | 0.008* |
| **Extremity changes** | 0.429 | 0.334 | 1.656 | 1.536 | 0.799-2.954 | 0.198 |
| **jaundice** | 0.038 | 0.744 | 0.003 | 1.039 | 0.242-4.462 | 0.959 |
| **Tachypnea** | 1.615 | 0.619 | 6.800 | 5.028 | 1.494-16.926 | 0.009* |
| **Irritability** | 0.276 | 0.394 | 0.491 | 1.318 | 0.609-2.854 | 0.483 |
| **Aseptic encephalitis** | 0.312 | 0.642 | 0.236 | 1.366 | 0.388-4.808 | 0.627 |
| **KD shock syndrome** | -1.416 | 1.251 | 1.281 | 0.243 | 0.021-2.817 | 0.258 |
| **Hemoglobin** | -0.052 | 0.011 | 21.907 | 0.950 | 0.929-0.970 | <0.001* |
| **CRP** | 0.002 | 0.002 | 0.513 | 1.002 | 0.997-1.006 | 0.474 |
| **AST** | <0.001 | 0.001 | 0.733 | 1.000 | 0.999-1.001 | 0.392 |
| **Bile acids** | 0.003 | 0.009 | 0.088 | 1.003 | 0.985-1.020 | 0.766 |
| **Serum sodium** | -0.119 | 0.046 | 6.765 | 0.888 | 0.811-0.971 | 0.009* |
| **PNI** | -0.048 | 0.014 | 12.083 | 0.953 | 0.927-0.979 | 0.001* |

**Note:** *p < 0.05.

**Abbreviations:** AST, aspartate transaminase; CRP, C-reactive proteins; KD, Kawasaki disease; PNI, prognostic nutritional index.

**Table 4** Binary logistic regression analysis to evaluate risk factors for IVIG resistance in Model 4.

| **Variables** | **B** | **S.E.** | **Waldχ2** | **OR** | **95%CI** | **p value** |
| --- | --- | --- | --- | --- | --- | --- |
| **Days of IVIG at initiation** | -0.251 | 0.100 | 6.365 | 0.778 | 0.640-0.945 | 0.012* |
| **Extremity changes** | 0.426 | 0.333 | 1.630 | 1.530 | 0.796-2.941 | 0.202 |
| **jaundice** | 0.161 | 0.734 | 0.048 | 1.175 | 0.279-4.946 | 0.826 |
| **Tachypnea** | 1.584 | 0.612 | 6.693 | 4.874 | 1.468-16.182 | 0.010* |
| **Irritability** | 0.246 | 0.391 | 0.396 | 1.279 | 0.595-2.751 | 0.529 |
| **Aseptic encephalitis** | 0.422 | 0.639 | 0.435 | 1.525 | 0.435-5.338 | 0.509 |
| **KD shock syndrome** | -1.283 | 1.276 | 1.012 | 0.277 | 0.023-3.377 | 0.314 |
| **Hemoglobin** | -0.180 | 0.086 | 4.357 | 0.835 | 0.706-0.989 | 0.037* |
| **Lymphocytes** | -0.052 | 0.011 | 21.830 | 0.949 | 0.928-0.970 | <0.001* |
| **AST** | <0.001 | 0.001 | 0.436 | 1.000 | 0.999-1.001 | 0.509 |
| **Bile acids** | 0.003 | 0.009 | 0.139 | 1.003 | 0.986-1.021 | 0.709 |
| **Serum sodium** | -0.126 | 0.046 | 7.600 | 0.882 | 0.806-0.964 | 0.006* |
| **CAR** | 0.146 | 0.064 | 5.163 | 1.157 | 1.020-1.312 | 0.023* |

**Note:** *p < 0.05.

**Abbreviations:** AST, aspartate transaminase; KD, Kawasaki disease; CAR, C-reactive protein to albumin ratio.

**Table 5** Binary logistic regression analysis to evaluate risk factors for IVIG resistance in Model 5.

| **Variables** | **B** | **S.E.** | **Waldχ2** | **OR** | **95%CI** | **p value** |
| --- | --- | --- | --- | --- | --- | --- |
| **Days of IVIG at initiation** | -0.266 | 0.102 | 6.845 | 0.767 | 0.628-0.935 | 0.009* |
| **Extremity changes** | 0.389 | 0.335 | 1.347 | 1.475 | 0.765-2.844 | 0.202 |
| **jaundice** | 0.079 | 0.759 | 0.011 | 1.082 | 0.244-4.794 | 0.917 |
| **Tachypnea** | 1.503 | 0.636 | 5.575 | 4.494 | 1.291-15.644 | 0.018* |
| **Irritability** | 0.232 | 0.397 | 0.341 | 1.261 | 0.579-2.747 | 0.559 |
| **Aseptic encephalitis** | 0.330 | 0.662 | 0.249 | 1.391 | 0.380-5.287 | 0.618 |
| **KD shock syndrome** | -1.618 | 1.247 | 1.685 | 0.198 | 0.017-2.282 | 0.194 |
| **Lymphocytes** | -0.056 | 0.095 | 0.345 | 0.946 | 0.786-1.139 | 0.557 |
| **Hemoglobin** | -0.053 | 0.011 | 22.443 | 0.949 | 0.928-0.970 | <0.001* |
| **CRP** | 0.001 | 0.002 | 0.104 | 1.001 | 0.996-1.005 | 0.747 |
| **AST** | 0.001 | 0.001 | 1.050 | 1.001 | 1.000-1.002 | 0.305 |
| **Bile acids** | 0.002 | 0.009 | 0.049 | 1.002 | 0.984-1.020 | 0.825 |
| **Serum sodium** | -0.117 | 0.046 | 6.421 | 0.890 | 0.813-0.974 | 0.011* |
| **NPAR** | 0.819 | 0.284 | 8.302 | 2.269 | 1.300-3.962 | 0.004* |

**Note:** *p < 0.05.

**Abbreviations:** AST, aspartate transaminase; KD, Kawasaki disease; NPAR, neutrophil percentage-to-albumin ratio.

**Table 6** Binary logistic regression analysis to evaluate risk factors for IVIG resistance in all Models

| **Model** | **Variables** | **B** | **S.E.** | **Waldχ2** | **OR** | **95%CI** | ***p* value** |
| --- | --- | --- | --- | --- | --- | --- | --- |
| **Base Model** | Days of IVIG at initiation | -0.287 | 0.095 | 9.043 | 0.751 | 0.622-0.905 | 0.003 |
|  | Tachypnea | 1.933 | 0.517 | 13.986 | 6.908 | 2.509-19.023 | <0.001 |
|  | Hemoglobin | -0.055 | 0.010 | 28.929 | 0.946 | 0.927-0.966 | <0.001 |
|  | Serum sodium | -0.156 | 0.041 | 14.562 | 0.855 | 0.789-0.927 | <0.001 |
| **Model 1** | Days of IVIG at initiation | -0.274 | .097 | 8.052 | 0.760 | 0.629-0.919 | 0.005 |
|  | Tachypnea | 1.833 | .535 | 11.756 | 6.253 | 2.193-17.831 | 0.001 |
|  | Hemoglobin | -0.051 | .011 | 23.748 | 0.950 | 0.930-0.970 | <0.001 |
|  | Serum sodium | -0.131 | .042 | 9.896 | 0.877 | 0.808-0.952 | 0.002 |
|  | CALLY index | -2.632 | 1.062 | 6.143 | 0.072 | 0.009-0.577 | 0.013 |
| **Model 2** | Days of IVIG at initiation | -0.284 | 0.098 | 8.429 | 0.752 | 0.621-0.912 | 0.004 |
|  | Tachypnea | 1.759 | 0.535 | 10.823 | 5.807 | 2.036-16.563 | 0.001 |
|  | Hemoglobin | -0.057 | 0.010 | 30.531 | 0.944 | 0.925-0.964 | <0.001 |
|  | Serum sodium | -0.143 | 0.042 | 11.408 | 0.866 | 0.797-0.942 | 0.001 |
|  | NAR | 1.804 | 0.678 | 7.089 | 6.073 | 1.610-22.916 | 0.008 |
| **Model 3** | Days of IVIG at initiation | -0.286 | 0.098 | 8.433 | 0.752 | 0.620-0.911 | 0.004 |
|  | Tachypnea | 1.539 | 0.545 | 7.968 | 4.660 | 1.601-13.567 | 0.005 |
|  | Hemoglobin | -0.053 | 0.011 | 24.920 | 0.948 | 0.928-0.968 | <0.001 |
|  | Serum sodium | -0.120 | 0.043 | 7.816 | 0.887 | 0.815-0.965 | 0.005 |
|  | PNI | -.051 | .013 | 15.717 | 0.950 | 0.927-0.975 | <0.001 |
| **Model 3** | Days of IVIG at initiation | -0.287 | 0.097 | 8.717 | 0.751 | 0.620-0.908 | 0.003 |
|  | Tachypnea | 1.577 | .547 | 8.303 | 4.839 | 1.656-14.141 | 0.004 |
|  | Hemoglobin | -0.049 | 0.011 | 20.725 | 0.953 | 0.933-0.973 | <0.001 |
|  | Serum sodium | -0.144 | 0.042 | 11.748 | 0.866 | 0.798-0.940 | 0.001 |
|  | CAR | 0.188 | 0.061 | 9.377 | 1.207 | 1.070-1.361 | 0.002 |
| **Model 4** | Days of IVIG at initiation | -0.287 | 0.099 | 8.347 | 0.750 | 0.618-0.912 | 0.004 |
|  | Tachypnea | 1.301 | 0.569 | 5.227 | 3.674 | 1.204-11.209 | 0.022 |
|  | Hemoglobin | -0.052 | 0.011 | 24.004 | 0.949 | 0.930-0.969 | <0.001 |
|  | Serum sodium | -0.116 | 0.044 | 6.942 | 0.891 | 0.817-0.971 | 0.008 |
|  | NPAR | 0.930 | 0.220 | 17.949 | 2.535 | 1.648-3.898 | <0.001 |
| **Model 5** | Days of IVIG at initiation | -0.286 | 0.098 | 8.433 | 0.752 | 0.620-0.911 | 0.004 |
|  | Tachypnea | 1.539 | 0.545 | 7.968 | 4.660 | 1.601-13.567 | 0.005 |
|  | Hemoglobin | -0.053 | 0.011 | 24.920 | 0.948 | 0.928-0.968 | <0.001 |
|  | Serum sodium | -0.120 | 0.043 | 7.816 | 0.887 | 0.815-0.965 | 0.005 |
|  | PNI | -.051 | .013 | 15.717 | 0.950 | 0.927-0.975 | <0.001 |

**Abbreviations:** CI, confidence interval; CALLY, C-reactive protein-albumin-lymphocyte; CAR, C-reactive protein to albumin ratio; NAR, neutrophil-to-albumin ratio; NPAR, neutrophil percentage-to-albumin ratio; PNI, prognostic nutritional index.

**Table 7** Area under the curve of variables in Model 1.

| **Variables** | **AUC** | **95%CI** | **p value** |
| --- | --- | --- | --- |
| **Days of IVIG at initiation** | 0.611 | 0.543-0.679 | 0.001 |
| **Hemoglobin** | 0.705 | 0.640-0.770 | <0.001 |
| **Serum sodium** | 0.668 | 0.604-0.731 | <0.001 |
| **CALLY** | 0.725 | 0.664-0.785 | <0.001 |

**Abbreviations:** AUC, area under curve; CI, confidence interval; CALLY, C-reactive protein-albumin-lymphocyte.

|  |
| --- |

**Table 8** Area under the curve of variables in Model 2.

| **Variables** | **AUC** | **95%CI** | **p value** |
| --- | --- | --- | --- |
| **Days of IVIG at initiation** | 0.611 | 0.543-0.679 | 0.001 |
| **Lymphocytes** | 0.652 | 0.580-0.724 | <0.001 |
| **Hemoglobin** | 0.705 | 0.640-0.770 | <0.001 |
| **Serum sodium** | 0.668 | 0.604-0.731 | <0.001 |
| **CAR** | 0.700 | 0.637-0.763 | <0.001 |

**Abbreviations:** AUC, area under curve; CI, confidence interval; CAR, C-reactive protein to albumin ratio.

**Table 9** Area under the curve of variables in Model 3.

| **Variables** | **AUC** | **95%CI** | **p value** |
| --- | --- | --- | --- |
| **Days of IVIG at initiation** | 0.611 | 0.543-0.679 | 0.001 |
| **Lymphocytes** | 0.652 | 0.580-0.724 | <0.001 |
| **Hemoglobin** | 0.705 | 0.640-0.770 | <0.001 |
| **Serum sodium** | 0.668 | 0.604-0.731 | <0.001 |
| **NAR** | 0.580 | 0.506-0.654 | 0.038 |

**Abbreviations:** AUC, area under curve; CI, confidence interval; NAR, neutrophil-to-albumin ratio.

**Table 10** Area under the curve of variables in Model 4.

| **Variables** | **AUC** | **95%CI** | **p value** |
| --- | --- | --- | --- |
| **Days of IVIG at initiation** | 0.611 | 0.543-0.679 | 0.001 |
| **Lymphocytes** | 0.652 | 0.580-0.724 | <0.001 |
| **Hemoglobin** | 0.705 | 0.640-0.770 | <0.001 |
| **Serum sodium** | 0.668 | 0.604-0.731 | <0.001 |
| **NPAR** | 0.717 | 0.651-0.784 | <0.001 |

**Abbreviations:** AUC, area under curve; CI, confidence interval; NPAR, neutrophil percentage-to-albumin ratio.

**Table 11** Area under the curve of variables in Model 5.

| **Variables** | **AUC** | **95%CI** | **p value** |
| --- | --- | --- | --- |
| **Days of IVIG at initiation** | 0.611 | 0.543-0.679 | 0.001 |
| **Hemoglobin** | 0.705 | 0.640-0.770 | <0.001 |
| **Serum sodium** | 0.668 | 0.604-0.731 | <0.001 |
| **PNI** | 0.712 | 0.643-0.788 | <0.001 |

**Abbreviations:** AUC, area under curve; CI, confidence interval; PNI, prognostic nutritional index.
